# Incidence and risk factors of opportunistic infections after autologous stem cell transplantation: a nationwide, population-based cohort study in Korea

**DOI:** 10.1038/s41598-023-27465-y

**Published:** 2023-02-13

**Authors:** Da Jung Kim, Seri Jeong, Seom Gim Kong, Sangjin Lee, Sung-Nam Lim, Sung Yong Oh, Young Rok Do, Won Sik Lee, Mark Hong Lee, Sung Hwa Bae, Se Hyung Kim, Min Kyoung Kim, Ho Sup Lee, Consortium for Improving Survival of Lymphoma (CISL)

**Affiliations:** 1 Department of Internal Medicine, Kosin University College of Medicine, 34 Amnam-Dong, Seo-Gu, Busan, 49267 South Korea; 2 Department of Laboratory Medicine, Kangnam Sacred Heart Hospital, Hallym University College of Medicine, Seoul, 07441 South Korea; 3 Department of Pediatrics, Kosin University College of Medicine, Busan, 49267 Korea; 4 Graduate School, Department of Statistics, Pusan National University, Busan, 46241 Korea; 5 Department of Internal Medicine, Haeundae Paik Hospital, College of Medicine Inje University, Busan, 48108 Korea; 6 Department of Internal Medicine, Dong-A University College of Medicine, Busan, 49201 Korea; 7 Division of Hematology-Oncology, Department of Medicine, Dongsan Medical Center, Keimyung University, Daegu, 41931 Korea; 8 Department of Internal Medicine, Busan Paik Hospital, College of Medicine Inje University, Busan, 47392 South Korea; 9 Division of Hematology-Oncology, Department of Internal Medicine, Konkuk University Medical Center, Konkuk University School of Medicine, Seoul, 05030 South Korea; 10 Department of Internal Medicine, Daegu Catholic University Medical Center, Daegu, 42472 South Korea; 11 Department of Internal Medicine, Soonchunhyang University Bucheon Hospital, Bucheon, 14584 South Korea; 12 Department of Hematology-Oncology, Yeungnam University Medical Center, Yeungnam University School of Medicine, Daegu, 42415 South Korea

**Keywords:** Lymphoma, Myeloma, Preventive medicine

## Abstract

Several guidelines classify autologous stem cell transplantation (ASCT) as a low to intermediate risk group for infection. In a nationwide population-based study, using the Korean Health Insurance Review and Assessment Service database, patients with lymphoma and multiple myeloma (MM) who underwent ASCT from 2002 to 2016 were retrospectively analyzed. Cumulative incidence rates (CIRs) and risk factors of opportunistic infections were investigated. CIRs of fungal, Varicella zoster virus (VZV), cytomegalovirus (CMV), and *Pneumocystis jirovecii* infections in lymphoma were 7.9%, 16.0%, 7.4%, and 5.1%, respectively, and CIRs in MM were 6.3%, 19.1%, 4.2%, and 5.6%, respectively. Fungal infection was significantly higher in patients with previous infection (Hazard ratio (HR) 2.003, p = 0.005) in lymphoma. Incidence of CMV infection was significantly higher in patients with prior CMV infection: HR 4.920, *p* < 0.001 (lymphoma); HR 3.022, *p* = 0.030 (MM). VZV infection was significantly lower in patients receiving prophylaxis: HR 0.082, *p* < 0.001 (lymphoma); HR 0.096, *p* < 0.001 (MM). For *P. jirovecii* infection, busulfex and melphalan conditioning (HR 1.875, *p* = 0.032) and previous *P. jirovecii* infection (HR 4.810, *p* < 0.001) had a higher incidence in MM. Patients who underwent ASCT should receive VZV prophylaxis and prophylaxis for fungal and *P. jirovecii* may be considered in patients with previous same infection.

## Introduction

Autologous hematopoietic stem cell transplantation (ASCT) is a well-established treatment option for hematologic malignancies, especially malignant lymphoma and multiple myeloma (MM). With the advances in medicine, the success rate of ASCT has improved; however, infection is still an important complication related to the prognosis of ASCT and can affect the quality of life and long-term survival in the early and late phases of transplantation^1–3^. Therefore, various prophylaxes have been used to reduce these infections. However, compared with allogeneic hematopoietic stem cell transplantation, ASCT has a lower risk of infection; therefore, few studies related to infection have been conducted.

According to the National Comprehensive Cancer Network (NCCN) guideline (version 1. 2021), ASCT is in the intermediate-risk group for overall infections. Based on this risk, for fungal infections, when mucositis is present, prophylaxis with fluconazole or echinocandin is recommended until recovery from neutropenia (category 1, for which there is a high level of evidence), and prophylaxis is not recommended without mucositis (category 2 B, where there is a lower level of evidence but no uniform consensus)^4^. Likewise, in the European Conference on Infections in Leukemia (ECIL) guideline, patients undergoing ASCT, for whatever underlying condition, are at a low risk of invasive fungal disease. Therefore primary antifungal propylaxis is not recommended, although fluconazole should be considered to prevent mucosal Candida infection during the neutropenic phase^5^. For Varicella zoster virus (VZV), it is recommended that acyclovir, famciclovir and valacyclovir be maintained for 6–12 months after the transplantation (category 2A, for which there is a lower level of evidence but uniform consensus) in NCCN guideline^4^. In both guidelines, cytomegalovirus (CMV) prophylaxis is not recommended as ASCT is not associated with the risk of CMV infection^4,6^. For *Pneumocystis jirovecii *(*P. jirovecii*), the NCCN guideline recommends prophylaxis with trimethoprim/sulfamethoxazole (category 1) or atovaquone, pentamidine, dapsone (category 2A) for 3–6 months after transplantation (Category 2B)^4^, but the ECIL guideline does not recommend prophylaxis^7^.

Even the two guidelines recommend differently for same infection, there are few reports on the incidence rate of opportunistic infections after ASCT in the literature^8–11^. In addition, most clinicians recognize the risk of infection and perform prophylaxis before engraftment, but for post-engraftment infections, prophylaxis is empirically conducted according to the opinion of the clinicians.

In this study, we investigated the incidence and risk factors of fungal and viral infections after ASCT in patients with lymphoma and MM using big data especially in post-engraftment period. Based on these data, we would like to consider the effectiveness of prophylactic antifungal and antiviral agents in clinical practice.

## Methods

### Data source

We used the nationwide and population-based Health Insurance Review and Assessment Service database, which is based on data from the universal health insurance system run by the Korean government. Since the National Health Insurance is the only public medical insurance system in Korea, the program covers the entire Korean population. The database includes patient demographics, principal diagnoses, and comorbidities based on the International Classification of Diseases, 10th Revision, prescription codes, and special procedures such as transplantation for both outpatients and inpatients. The National Health Insurance Service provide these extensive data for use in research after the approval process. The independent Institutional Review Board of Kosin University Gospel Hospital approved this study and granted a waiver of informed consent from the study participants because anonymity of personal information was maintained (KUGH 2017-11-026). The data acquisition number for the National Health Insurance Sharing Service was REQ0000016684. All methods were carried out in accordance with relevant guidelines and regulations along with the approval.

### Patient selection

From January 2002 to December 2016, of the patients registered with malignant lymphoma and MM who were over 18 years of age, those who underwent ASCT were enrolled. Patients who underwent allogeneic hematopoietic stem cell transplantation after ASCT were excluded.

Infections were limited to cases that occurred at least 30 days after ASCT. Fungal infections were defined as invasive candidiasis and aspergillosis, or cases in which antifungal agents such as intravenous (IV) amphotericin B, IV liposomal amphotericin B, IV caspofungin, IV itraconazole, IV voriconazole, and IV fluconazole were prescribed for more than 7 days. Patients with oral candidiasis were excluded from this study. Viral infection was defined as VZV reactivation, CMV viremia or disease, and was defined as IV acyclovir, per oral (po) famciclovir for more than 5 days, and IV ganciclovir and valganciclovir for more than 7 days. Herpes simplex virus and Epstein–Barr virus infections were excluded. *P. jiroveciii* infection was limited to cases in which IV trimethoprim/sulfamethoxazole was administered for more than 7 days and when *P. jiroveciii* infection or pneumonia was diagnosed.

Prophylactic drugs were defined as drugs used in clinical practice, based on the National Comprehensive Cancer Network guidelines. The antifungal prophylactic agents were po fluconazole, and anti*-P. jirovecii* prophylaxis was detected by po trimethoprim/sulfamethoxazole. In the case of antiviral therapy, VZV prophylaxis included po acyclovir and po valaciclovir. However, prophylaxis for CMV infection was not analyzed because there was no meaningful prophylactic therapy available at the time of analysis. Prophylaxis included cases in which each prophylactic agent was administered for ≥ 30 days.

### Statistical analysis

We evaluated the cumulative incidence and risk factors of opportunistic infections after ASCT in patients with lymphoma and MM in Korea from 2002 to 2016. Because lymphoma and MM are different diseases and the intensity of conditioning is different, the incidence and risk factors of both diseases were analyzed. Incidence was calculated using the number of individuals with opportunistic infections as the numerator, and the patients who underwent ASCT as the denominator. The cumulative incidence rate (CIR) was reported at a specific time after transplantation (6 months, 12 months, and 5 years) using a landmark approach. The cumulative incidence was estimated using the Kaplan–Meier method. The strength of the association between the variables and opportunistic infections was assessed using Cox proportional hazards regression analysis. We used maximally selected log-rank statistics in the maxstat function of R software (version 3.4.4) to identify the optimal threshold to assess the cumulative incidence for age. We selected optimal age cutoffs of 45 years for lymphoma and 55 years for MM. Logistic regression analysis was used for multivariate analysis. Statistical analyses were performed using the R statistical software (version 3.4.4; R Foundation for Statistical Computing) and SAS statistical analysis software (version 9.4; SAS Institute Inc., Cary, NC, USA). Statistical significance was set at *P* < 0.05.

## Results

### Patients characteristics

The characteristics of the 6516 patients who underwent ASCT for lymphoma (n = 3236) and MM (n = 3280) are described in Table 1. The median age was 48 years (range 18–70) for lymphoma and 56 years (range 18–68) for myeloma at the time of transplantation, and 62.5% (n = 2022) and 55.9% (n = 1834) of the patients were men with lymphoma and myeloma, respectively.

**Table 1:** Patient characteristics.

Value (%)	Lymphoma (n = 3236) (%)		Multiple myeloma (n = 3280) (%)
**Age, years**			
Median (range)	48 (18–70)		56 (18–68)
**Sex (%)**			
Male	2022 (62.5)		1834 (55.9)
Female	1214 (37.5)		1446 (44.1)
**Diagnosis (%)**			
DLBCL/Burkitt lymphoma	1672 (51.7)		
Indolent B cell lymphoma	479 (14.8)		
T cell lymphoma	671 (20.7)		
Others (Hodgkin’s disease, etc.)	414 (12.8)		
**Year of transplantation (%)**			
2002–2006	520 (16.1)		468 (14.3)
2007–2011	1100 (34.0)		1059 (32.3)
2012–2016	1616 (49.9)		1753 (53.4)
**Conditioning regimen (%)**			
BuCyE	1084 (33.5)	Melphalan only	2880 (87.8)
BuMelE	849 (26.2)	BuMel	237 (7.2)
Others	1303 (40.3)	Others	163 (5.0)
**Comorbidity (%)**			
< 2	2898 (89.6)		2701 (82.3)
≥ 2	338 (10.4)		579 (17.7)
**Pre-transplantation infection (%)**			
Fungal	141 (4.4)		78 (2.4)
VZV	445 (13.8)		691 (21.1)
CMV	35 (1.1)		40 (1.2)
*P. jiroveciii* infection	60 (1.9)		31 (0.9)
**Prophylaxis (%)**			
Fungal	208 (6.4)		205 (6.3)
VZV	375 (11.6)		540 (16.5)
*P. jiroveciii*	187 (5.8)		406 (12.4)
**Post-transplantation infection (%)**			
Fungal	221 (6.8)		161 (4.9)
VZV	461 (14.2)		531 (16.2)
CMV	211 (6.5)		117 (3.6)
*P. jiroveciii*	143 (4.4)		143 (4.4)

*DLBCL* Diffuse large B cell lymphoma, *BuCyE* Busulfan and Cyclophosphamide and Etoposide, *BuMelE* Busulfan and melphalan and Etoposide, *P. jiroveciii* *Pneumocystitis jiroveciii*, *VZV* Varicella zoster virus, *CMV* cytomegalovirus.

Of the patients with lymphoma, 51.7% of were diagnosed with diffuse large B-cell lymphoma or Burkitt lymphoma. There were 141 (4.4%), 445 (13.8%), 35 (1.1%), and 60 (1.9%) patients with fungal, VZV, CMV, and *P. jirovecii* infection prior to transplantation, respectively. The number of patients who received preventive treatment for fungal, VZV, and *P. jirovecii* infections was 208 (6.4%), 375 (11.6%), and 187 (5.8%), respectively.

For MM, 78 (2.4%), 691 (21.1%), 40 (1.2%), and 31 (0.9%) patients had a history of fungal, VZV, CMV, and *P. jirovecii* infections, respectively, prior to transplantation. A total of 205 (6.3%), 540 (16.5%), and 406 (12.4%) patients received prophylaxis against fungal, VZV, and *P. jirovecii* infections, respectively.

Compared to 2002–2006, the number of transplantations increased from 520 to 1616 for lymphoma and from 468 to 1753 for MM, in 2012–2016.

### Incidence of opportunistic infections

**Figure 1:**
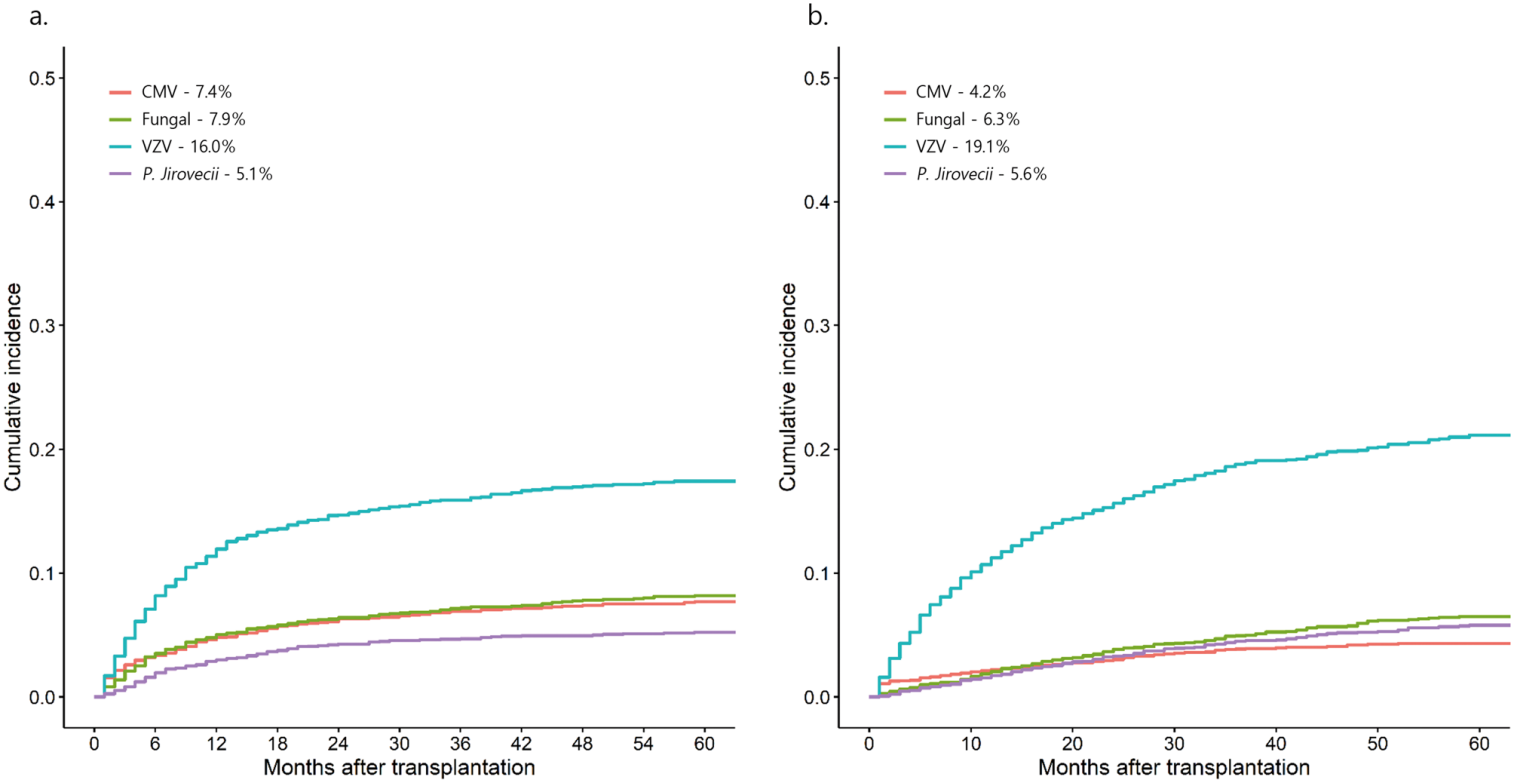
Cumulative incidence rates (CIRs) of opportunistic infections. (**A**) Lymphoma. (**B**) Multiple myeloma. (**A**) The CIRs at 6 months of fungal, Varicella zoster virus (VZV), Cytomegalovirus (CMV), *and Pneumocystis jirovecii* (*P. jiroveciii)* infection was 3.4%, 7.8%, 3.3% and 1.9%, respectively. The CIRs at 12 months was 4.9%, 11.3%, 4.7% and 2.9%. (**B**) The CIRs at 6 months of fungal, VZV, CMV, and *P. jiroveciii* infection was 1.1%, 7.2%, 1.6% and 0.8%, respectively. The CIRs at 12 months was 2.0%, 10.6%, 2.2% and 1.7%.

For lymphoma, 221 (6.8%), 461 (14.2%), 211 (6.5%), and 143 (4.4%) patients were infected with the fungi, VZV, CMV, and *P. jirovecii* pneumonia (PJP), respectively, and 161 (4.9%), 531 (16.2%), 117 (3.6%), and 143 (4.4%) patients with MM, respectively, after transplantation. (Table 1) The CIRs at 6 months of fungal, VZV, CMV, and *P. jiroveciii* infection in lymphoma were 3.4%, 7.8%, 3.3% and 1.9%, respectively, and the CIRs in MM were 1.1%, 7.2%, 1.6% and 0.8%, respectively. The CIRs at 12 months for fungal, VZV, CMV, and *P. jirovecii* infection in lymphoma were 4.9%, 11.3%, 4.7%, and 2.9%, respectively, and the CIRs in MM were 2.0%, 10.6%, 2.2%, and 1.7%, respectively. The CIRs at 5 years for fungal, VZV, CMV, and *P. jirovecii* infection in lymphoma were 7.9%, 16.0%, 7.4%, and 5.1%, respectively, and the CIRs in MM were 6.3%, 19.1%, 4.2%, and 5.6%, respectively (Fig. 1).

### Analysis of risk factor for opportunistic infections

The results of the univariate and multivariate analyses of the associations with opportunistic infections in lymphoma and MM are shown in Tables 2 and 3. Multivariate analysis showed that the factors affecting fungal infection were the year of transplantation (hazard ratio (HR) = 2.330 [95% confidence interval (95% CI) 1.485–3.658]; *p* < 0.001 in lymphoma patients, HR = 2.650 [95% CI 1.519–4.623]; *p* = 0.001 in MM patients) and previous fungal infection history (HR = 2.003 [95% CI 1.236–3.244]; *p* = 0.005 in lymphoma patients only). For patients with CMV infection, in the multivariate analysis, the following factors showed a significantly higher incidence of infection: year of transplantation (HR = 2.752 [95% CI 1.689–4.484]; *p* < 0.001 in lymphoma patients only) and previous CMV infection history (HR = 4.920 [95% CI 2.552–9.600]; *p* < 0.001 in lymphoma patients and HR = 3.022 [95% CI 1.115–8.191]; *p* = 0.030 in MM patients). Multivariate analysis showed that other conditioning regimens (HR = 1.349 [95% CI 1.081–1.682]; *p* = 0.008 in lymphoma patients only) and prophylaxis (HR = 0.082 [95% CI 0.034–0.197]; *p* < 0.001 in lymphoma and HR = 0.096 [95% CI 0.053–0.175]; *p* < 0.001 in MM) were independent predictive factors for VZV reactivation. Based on multivariate analysis for *P. jirovecii* infection, the year of transplantation (HR = 2.552 [95% CI 1.439–4.526]; *p* = 0.001 in lymphoma and HR = 3.800 [95% CI 1.801–8.021]; *p* < 0.001 in MM) had a higher incidence. For MM patients, busulfan and melphalan conditioning (HR = 1.875 [95% CI 1.055–3.333]; *p* = 0.032), previous *P. jirovecii* infection (HR = 4.810 [95% CI 2.120–10.913]; *p* < 0.001) and prophylaxis (HR = 1.564 [95% CI 1.040–2.352]; *p* = 0.032) had a higher incidence.

**Table 2:** Univariate and multivariate Cox analysis for atypical infection in lymphoma.

Value	Fungal				CMV				VZV				*Pneumocystis jirovecii*			
	Univariate HR (95% CI)	p value	Multivariate HR (95% CI)	p value	Univariate HR (95% CI)	p value	Multivariate HR (95% CI)	p value	Univariate HR (95% CI)	p value	Multivariate HR (95% CI)	p value	Univariate HR (95% CI)	p value	Multivariate HR (95% CI)	p value
**Age, years**																
≤ 45																
> 45	1.017 (0.779–1.328)	0.901			0.956 (0.729–1.255)	0.747			1.147 (0.952–1.383)	0.149			0.804 (0.579–1.116)	0.192		
**Sex**																
Male																
Female	0.906 (0.688–1.194)	0.485			0.999 (0.756–1.320)	0.992			0.970 (0.803–1.172)	0.751			0.709 (0.496–1.014)	0.060		
**Diagnosis**																
Indolent B cell lymphoma																
DLBCL / Burkitt lymphoma	1.176 (0.770–1.796)	0.453			1.223 (0.802–1.864)	0.350			1.079 (0.820–1.421)	0.586			1.063 (0.638–1.771)	0.815		
T cell lymphoma	1.649 (1.044–2.604)	0.032	1.361 (1.008–1.837)	0.044	1.557 (0.982–2.467)	0.060			1.275 (0.937–1.734)	0.122			1.716 (0.998–2.951)	0.051		
Others (HD, etc.)	1.403 (0.841–2.341)	0.195			0.912 (0.515–1.612)	0.750			0.991 (0.692–1.419)	0.959			0.995 (0.512–1.934)	0.988		
**Year of transplantation**																
2002 ~ 2006																
2007 ~ 2011	1.669 (1.050–2.653)	0.030	1.653 (1.040–2.629)	0.034	1.844 (1.113–3.055)	0.017	1.812 (1.093–3.002)	0.021	0.892 (0.695–1.144)	0.368			1.744 (0.966–3.151)	0.065	1.744 (0.966–3.151)	0.065
2012 ~ 2016	2.342 (1.492–3.676)	< 0.001	2.330 (1.485–3.658)	< 0.001	2.815 (1.728–4.585)	< 0.001	2.752 (1.689–4.484)	< 0.001	0.778 (0.606–0.998)	0.048			2.552 (1.439–4.526)	0.001	2.552 (1.439–4.526)	0.001
**Conditioning regimen**																
BuCyE																
BuMelE	1.177 (0.853–1.624)	0.321			1.292 (0.923–1.809)	0.136			1.225 (0.953–1.575)	0.113	1.194 (0.929–1.536)	0.166	1.571 (1.039–2.375)	0.032	1.295 (0.902–1.859)	0.160
Others	0.732 (0.531–1.008)	0.056			0.878 (0.632–1.219)	0.438			1.443 (1.157–1.799)	0.001	1.349 (1.081–1.682)	0.008	1.004 (0.667–1.512)	0.984		
**Comorbidity**																
≥ 2 comorbidity	0.949 (0.616–1.461)	0.811			0.761 (0.469–1.233)	0.267			0.870 (0.637–1.187)	0.378			0.544 (0.277–1.068)	0.077		
Previous infection history	2.042 (1.261–3.307)	0.003	2.003 (1.236–3.244)	0.005	5.288 (2.712–10.314)	< 0.001	4.920 (2.552–9.600)	< 0.001	0.837 (0.629–1.114)	0.222			1.267 (0.404–3.977)	0.685		
Prophylaxis	0.743 (0.405–1.362)	0.336							0.079 (0.033–0.191)	< 0.001	0.082 (0.034–0.197)	< 0.001	0.678 (0.299–1.535)	0.351		

*CMV* Cytomegalo virus, *VZV* Varicella zoster virus, *HR* hazard ratio, *CI* confidence interval, *DLBCL* diffuse large B cell lymphoma, *HD* Hodgkin’s disease, *BuCyE* Busulfan and Cyclophosphamide and Etoposide, *BuMelE* Busulfan and Melphalan and Etoposide;

**Table 3:** Univariate and multivariate Cox analysis for atypical infection in multiple myeloma.

Value	Fungal				CMV				VZV				*Pneumocystis jirovecii*			
	Univariate HR (95% CI)	p value	Multivariate HR (95% CI)	p value	Univariate HR (95% CI)	p value	Multivariate HR (95% CI)	p value	Univariate HR (95% CI)	p value	Multivariate HR (95% CI)	p value	Univariate HR (95% CI)	p value	Multivariate HR (95% CI)	p value
**Age, years**																
≤ 55																
> 55	0.816 (0.598–1.115)	0.202			0.695 (0.480–1.006)	0.054			0.861 (0.726–1.022)	0.087			1.024 (0.738–1.422)	0.887		
**Sex**																
Male																
Female	0.817 (0.596–1.120)	0.209			0.813 (0.561–1.178)	0.275			0.956 (0.805–1.136)	0.611			0.751 (0.536–1.054)	0.098		
**Year of transplantation**																
2002–2006																
2007–2011	1.877 (1.069–3.297)	0.028	1.877 (1.069–3.297)	0.028	1.227 (0.704–2.136)	0.470			0.905 (0.713–1.149)	0.413			3.740 (1.795–7.789)	< 0.001	3.560 (1.706–7.430)	0.001
2012–2016	2.650 (1.519–4.623)	0.001	2.650 (1.519–4.623)	0.001	1.163 (0.670–2.018)	0.591			0.793 (0.625–1.006)	0.056			4.210 (2.013–8.803)	< 0.001	3.800 (1.801–8.021)	< 0.001
**Conditioning regimen**																
Melphalan only																
BuMel	1.659 (0.938–2.933)	0.082			0.872 (0.383–1.988)	0.745			0.897 (0.613–1.314)	0.578			2.142 (1.230–3.732)	0.007	1.875 (1.055–3.333)	0.032
Others	0.493 (0.157–1.547)	0.225			0.859 (0.316–2.331)	0.765			0.961 (0.607–1.519)	0.863			0.789 (0.291–2.135)	0.640	1.011 (0.372–2.743)	0.984
**Comorbidity**																
≥ 2 comorbidity	0.835 (0.552–1.263)	0.393			0.837 (0.512–1.368)	0.477			0.902 (0.720–1.129)	0.369			1.354 (0.925–1.983)	0.119		
Previous infection history	1.495 (0.661–3.379)	0.334			3.017 (1.113–8.177)	0.030	3.022 (1.115–8.191)	0.030	1.043 (0.850–1.281)	0.686			4.883 (2.156–11.060)	< 0.001	4.810 (2.120–10.913)	< 0.001
Prophylaxis	1.459 (0.858–2.483)	0.163							0.096 (0.053–0.175)	< 0.001	0.096 (0.053–0.175)	< 0.001	1.616 (1.075–2.429)	0.021	1.564 (1.040–2.352)	0.032

*CMV* Cytomegalo virus, *VZV* Varicella zoster virus, *HR* hazard ratio, *CI* confidence interval, *BuMel* Busulfan and Melphalan;

## Discussion

ASCT is the standard treatment for malignant lymphoma and MM. Depending on the diagnosis, this procedure is indicated as frontline treatment and in other cases as salvage therapy^12,13^. Despite current improvements in supportive care, mortality and complications after ASCT remain important concerns. Although the morbidity and mortality of ASCT are lower than those of allogeneic transplantation, deaths still occur, mainly because of infectious complications^1,3^. In ASCT, most infections occur during neutropenia, usually about a month after transplantation. However, opportunistic infections occur after recovery from neutropenia, which affects the quality of life and survival of patients.

This is because reconstitution of bone marrow (BM) includes functional recovery of cellular interaction as well as simple numerical recovery of BM cellular elements^14^. Several studies have shown that immune modulation occurs in ASCT^14–16^. Despite the heterogeneity of diseases, age, pre-transplant treatment and high-dose conditioning regimens, lymphocyte subset analysis did not reveal any differences in immune reconstitution. All patients had a low CD4(+)/CD8(+) ratio during at least the first year post-transplantation, which was caused by a persistent increase in CD8(+) lymphocytes and reduction of CD4(+) lymphocytes, making the patients vulnerable to infections for a prolonged period time post-transplant^16^. Therefore, in the post-engraftment period, patients often take antibiotics, antifungal, and antiviral agents for weeks to months.

However, few studies have evaluated the incidence of opportunistic infections and the impact of prophylaxis on infections in ASCT, focusing on a large population, especially in the post-engraftment period. We conducted a nationwide population-based study to provide information regarding the incidence and risk factors of opportunistic infections in patients with lymphoma and MM after ASCT in the Korean population. In particular, we analyzed whether prophylactic therapy is effective and investigated infections after engraftment for a long-term period of up to 5 years.

Among ASCT recipients, VZV reactivation occurred in 20–43% of the patients and extended through the first year^17–21^. Erard et al. demonstrated the probability of reactivation of VZV in 8.2% of the patients who received low prophylactic doses of acyclovir for one year^22^. In patients who did not receive acyclovir or who took it for a shorter period (until the end of neutropenia), the rates increased to 21–25%^17,22^. In this study, VZV prophylaxis was found to be a significant predictive factor for VZV reactivation. (HR = 0.082 [95% CI 0.034–0.197]; *p* < 0.001 in lymphoma and HR = 0.096 [95% CI 0.053–0.175]; *p* < 0.001 in MM) As seen in several studies, the efficacy and safety of prophylaxis for VZV after SCT is well established, although there is poor consensus on the dose and duration of prophylaxis after ASCT^21^. In our study, since the CIR of VZV reactivation increased steeply up to 1 year (Fig. 1), we think it would be better to provide prophylaxis for 1 year. This result supports the duration of prophylaxis for VZV, according to the NCCN guideline. The cumulative dose of steroid was an independent risk factor for VZV reactivation after chemotherapy in lymphoma performed at our institution (HR = 7.717 [95% CI 3.814–18.703]; *p* < 0.001)^23^, but there is a limitation in that it is not possible to check the dose of steroid using big data. Previous VZV infection was also a significant risk factor^23^. However, due to the nature of this study, since the data were collected from the time of diagnosis of the disease, the infection before diagnosis of lymphoma or myeloma was not identified, which is considered a limitation of big data.

In large studies involving more than 1000 patients with ASCT, the incidence of invasive fungal infection was 1.2–1.5%^8–10^. In our data, the incidence of invasive fungal infection was higher than that in large-scale studies (6.8% in lymphoma, 4.9% in MM), because studies in other countries included only proven or probable cases. Our data included cases with empirical therapy, and there was a limitation in the insurance system in that one had to include a diagnostic code in order to prescribe antifungal agents. Therefore, the incidence in our study may have been overestimated. Fungal infection occurred more frequently in T cell lymphoma (HR = 1.361 [95% CI 1.008–1.837]; *p* = 0.044), probably because T cell lymphoma generally has a poorer prognosis than other type of lymphoma, with more common relapse or refractory state requiring further chemotherapy or steroid administration. As the rate of prophylaxis is low in fungal infections (6.3%), it is difficult to analyze the effectiveness of prophylaxis.

For CMV infection, the infection rate differs according to the diagnostic strategy. The incidence of CMV infection ranged from 17 to 33%, when prospectively monitored by antigenemia and/or viremia by PCR^24–27^. On the other hand, the frequencies were reported to range between 3 and 13% when applying a clinical-based strategy^28–33^. In our case, the diagnosis was based on clinical suspicion, and the results were similar to those of previous studies. In Korea, most transplantation centers introduce pre-emptive therapy rather than routine universal prevention because of the insurance coverage, cost–benefit ratio, and adverse drug reactions^34^. There is no effective prophylactic treatment for CMV in Korea; therefore, prophylaxis was not analyzed.

A large series in the 1990s reported an incidence rate for PJP of 1.4% in ASCT recipients^35^. Another study using a large stem cell transplantation registry showed that the incidence of PJP was 0.28% (n = 52 cases of 18,525 total)^11^. In previous studies, the incidence rate may have been underestimated because those with a history of PJP prior to transplantation were excluded. It is also possible that the incidence was overestimated in our study because we included patients without a pathological diagnosis.

In our study, fungal, CMV, and *P. jiroveciii* infections showed an increased risk in 2012–2016 compared to 2002–2006, which is thought to be because the number of transplants increased and more high-risk patients were transplanted. In fact, the median age of patients increases with the transplantation period. Compared to 2002–2006, in 2012–2016, it increased from 44 (range 18–65) to 50 (range 18–70) in lymphoma patients and from 54 (range 25–65) to 56 (range 18–68) in MM. In MM, the induction regimen including bortezomib has been available since October 2015, it is thought that bortezomib increased VZV infection in patients who underwent ASCT after 2016. Prior to transplantation, the same type of infection was a risk factor for all infections except VZV (Table 3). Therefore, we suggests that prophylaxis needs to be considered in patients with prior infection.

This study has several limitations. First, similar to other studies using big data, the results are based on physicians’ diagnoses without additional microbiological confirmation, possibly resulting in overestimation of the incidence. In particular, since the administration of a therapeutic agent is defined as infection, there is a possibility that empirical treatment will be included. However, in order to exclude empirical treatment as much as possible, the minimum duration of medication was defined, and considering Korean insurance system, it is thought that clinically unlikely cases were not included. In addition, because we used data from the Health Insurance Review and Assessment Service’s reimbursement system, we were unable to analyze drugs that were not reimbursed, such as VZV vaccine, voriconazole, atovaquone, pentamidine, and dapsone. The lack of detailed clinical information such as disease status (e.g., complete remission, partial remission or relapse etc.), laboratory data (WBC, lymphocyte count, etc.), previous or post-transplantation chemotherapy (maintenance or salvage chemotherapy) limits the analysis of various risk factors. Despite these limitations, it is a large-scale study covering the entire national population over 14 years in Korea and meaningful in that it evaluated the incidence of various opportunistic infections and the effectiveness of prophylactic therapy in the same disease group.

In conclusion, ASCT has a relatively low risk of infection compared to allogeneic hematopoietic stem cell transplantation because it does not accompany graft-versus-host disease and does not take immunosuppressants. However, since immune reconstitution is a process that occurs in both autologous and allogeneic stem cell transplantation, it is thought to be related to opportunistic infections in ASCT after engraftment. Based on the incidence and effectiveness of prophylaxis, patients undergoing ASCT should receive prophylaxis for VZV. In addition, prophylaxis for fungal infections and PJP needs to be considered in patients who have previously had an infection with the same organism. Further studies are required to determine the appropriate duration and dose of prophylaxis.

## Data Availability

All data generated or analyzed during this study are included in this published article (Tables and Figures) and available from the corresponding author on reasonable request. The additional raw data are available on request to the National Health Insurance Service, Korea.
